# Determinants, Screening, Prevention and Management of Obesity in Youth: New Evidence and Horizons

**DOI:** 10.3390/nu14163280

**Published:** 2022-08-11

**Authors:** Odysseas Androutsos, Evangelia Charmandari

**Affiliations:** 1 Laboratory of Clinical Nutrition and Dietetics, Department of Nutrition and Dietetics, School of Physical Education, Sport Science and Dietetics, University of Thessaly, 42132 Trikala, Greece; 2 Division of Endocrinology, Metabolism and Diabetes, First Department of Pediatrics, National and Kapodistrian University of Athens Medical School, ‘Aghia Sophia’ Children’s Hospital, 11527 Athens, Greece; 3 Division of Endocrinology and Metabolism, Center of Clinical, Experimental Surgery and Translational Research, Biomedical Research Foundation of the Academy of Athens, 11527 Athens, Greece

The prevalence of obesity has significantly increased over the last four decades worldwide. Obesity is associated with an increased prevalence of cardiometabolic disorders across the lifespan. The etiology of obesity has been attributed to demographic, socioeconomic, behavioral (e.g., unhealthy nutrition and low levels of physical activity), prenatal, perinatal, and clinical risk factors. However, their exact role, interplay, and mechanisms implicated in this process remain unclear. The trends of childhood obesity call for actions regarding the prevention and management of this disease early in life.

The Special Issue “Childhood Obesity: Nutrition and Lifestyle Determinants, Prevention and Management” of the journal *Nutrients* has collected original articles and reviews to advance the current knowledge on the role of lifestyle behaviors in the development of overweight and obesity, provide evidence about the nutritional habits of overweight and obese children and adolescents, and describe novel approaches for the screening, prevention, and management of obesity in youth. Towards achieving these goals, twenty articles were published in this Special Issue, and their main results are presented in the following paragraphs.

The studies by Sarintohe et al. and Thamrin et al. present new data regarding the prevalence of obesity in Indonesia [1,2]. Sarintohe et al. focused on adolescents (n = 411) attending private schools in Indonesia. According to the results, 36.3% of the participants were overweight, with the prevalence being significantly higher in boys compared to girls (47% vs. 24%), in urban compared to suburban areas (40.3% vs. 30.4%) and in adolescents who reported previous dieting at least once compared to those who never followed a restrictive diet plan (49.8% vs. 23.1%). Interestingly, boys living in urban areas were at higher risk of being overweight, suggesting that this specific group may need to be prioritized in future obesity-prevention measures. Similarly, Thamrin et al. showed that the prevalence of obesity was high in all the island clusters of Indonesia, which were included in their study. Different distributions of determinants of obesity were also recorded in these areas, highlighting the need for tailoring the policies and interventions to tackle the rising trends of obesity, accordingly.

Two more cross-sectional studies focused on the impact of the COVID-19 pandemic on children’s and adolescents’ lifestyle behaviors, well-being and body weight [3,4]. The COVEAT study by Androutsos et al. was conducted during the first lockdown implemented in Greece in April–May 2020 and examined its influence on 397 children’s and adolescents’ body weights and lifestyle behaviors. As expected, physical activity levels decreased, while sleep duration and screen time increased. Regarding the dietary behavior, children’s and adolescents’ consumption of fruit, fresh fruit juices, vegetables, dairy products, pasta, sweets, total snacks and breakfast increased. By contrast, fast-food consumption decreased. More than 1 out of 3 participants increased their body weight during the 2-month home confinement, which may be attributable to their increased consumption of certain foods/snacks and decreased level of physical activity. Similarly, the study by Morres et al. conducted a web-based survey with 950 adolescents during the second lockdown period implemented in Greece (November 2020–January 2021). This study showed that participants’ quality of life was below the WHO threshold for possible depressive symptoms. In addition, low levels of physical activity and eating behavior scores were recorded. Overall, the studies by Androutsos et al. and Morres et al. highlighted the negative impact of the COVID-19 lockdown measures on children’s and adolescents’ lifestyles, well-being and body weights, indicating a need to support healthy lifestyles in potential future lockdowns and similar circumstances of home confinement.

The studies by Usheva et al. and Hampson et al. shed more light on the determinants of obesity in early life [5,6,7]. More specifically, Usheva et al. examined the role of breastfeeding and complementary feeding in a large-scale European cohort (ToyBox-study). In total, 7554 children from six European countries (Belgium, Bulgaria, Germany, Greece, Poland and Spain) were included in the statistical analyses. The percentage of families who adhered to the WHO recommendation of exclusive breastfeeding for 6 months was particularly low (6.3%), while about half of the children breastfed for 4–6 months, thus indicating a need for public health actions to support breastfeeding in Europe. Moreover, the results of the two studies by Usheva et al. did not identify any significant association between breastfeeding or the timing of solid food introduction and obesity at preschool age, after adjusting for several potential confounders. Hampson et al. conducted a longitudinal study, using data from the “Southern California Mother’s Milk Study”, to investigate the impact of infant formulas made with added corn-syrup solids on Hispanic children’s eating behavior. At 2 years of follow-up, the consumption of this type of formula led to the development of greater food fussiness and reduced enjoyment of food compared to the observations recorded in breastfed children.

Guivarch et al. conducted a longitudinal study using data from the “EDEN mother-child cohort” to explore the determinants of obesity in toddlerhood [8]. More specifically, the authors examined the associations between an infant’s appetite (at months 4, 8, 12 and 24 of life) and genetic susceptibility to obesity with parental feeding practices. The study showed that the genetic susceptibility to obesity was not associated with parental feeding practices. Furthermore, the infant’s appetite was associated with restrictive feeding, while the parents of boys with high appetite in infancy more frequently used food to regulate their children’s emotions. Considering that parental feeding practices comprise a key determinant for children’s nutritional intakes and, consequently, their weight status, future obesity-prevention interventions should aim to improve them, possibly by targeting parents’ perceptions of their children’s appetites.

The narrative review by Mahmood et al. examined the associations of parents’ dietary behavior and practices with their children’s dietary behavior [9]. In total, 83 studies with data from 2–13-year-old children were included in this review and produced important results that are expected to be considered for future dietary interventions in youth. First, family meals seem to play a crucial role for modeling the dietary behavior of parents and their children, since it provides the time and opportunity to interact and exert control wherever needed. Secondly, parental encouragement and the avoidance of excessive pressure and restrictions against food consumption may support healthy eating and set a basis for the prevention of overconsumption and excessive weight gain in childhood and adolescence.

The cross-sectional study by Tani et al. included a sample of 5257 children aged 9–14 from Japan and examined whether the cooking skills of caregivers could influence children’s dietary intakes and weight status [10]. The results showed that caregivers with poor cooking skills cooked less frequently at home and that their children were less likely to consume vegetables frequently, and they were at high risk for being obese. These findings indicate a need to improve cooking skills as a strategy to promote healthy eating and prevent childhood obesity.

The aforementioned risk factors, along with other risk factors previously described in the scientific literature, contribute to the development of excessive weight gain and childhood obesity. The detrimental effect of obesity on health is largely attributed to the excessive body fat. The study by Orsso et al. aimed to describe the developmental trajectories of adipose tissue from intrauterine life to adolescence, and highlight the determinants of adiposity [11]. Different developmental phases were associated with different changes in body composition. The intrauterine factors influencing the development of adipose tissue included maternal health, maternal nutrition, exposure to toxins and genetic predisposition, while in infancy, feeding practices and the gut microbiome played significant roles. In puberty, sexual dimorphism in hormone secretion comprised the key determinant of adiposity and was accompanied by other risk factors, such as dietary behavior, the gut microbiome and immune cell function.

The short-term, negative effects of obesity on children’s health are well-known. The study by Zapata et al. aimed to describe the potential effect of obesity on Caucasian children’s and adolescents’ resting energy expenditure (REE) [12]. Both the REE and body composition of the participants were assessed using gold-standard techniques (i.e., indirect calorimetry and air-displacement plethysmography, respectively). According to the results of this study, REE was not influenced by obesity in children and adolescents after adjusting for fat-free mass (FFM). Another interesting finding was that this association remained insignificant in 8–10-year-old children, although a positive correlation between serum leptin and REE/FFM was recorded. These observations may be useful in the estimation of children’s and adolescents’ energy requirements, as well as in understanding of the impact of obesity on individuals’ health.

Previous studies have suggested that certain anthropometric indices widely used in clinical practice, such as the body mass index (BMI), are not sensitive enough for identifying children at high risk for developing cardiometabolic disorders. Therefore, the study by Chin et al. used data from the “Adolescent Nutrition and Health Survey” in Taiwan and the “Multilevel Risk Profiles for Adolescent Metabolic Syndrome Study” to test adipose indices that could identify adolescents with metabolic syndrome [13]. Only body-fat- and lipid-enhanced adiposity indicators could identify adolescents with metabolic syndrome, while the waist circumference in males and abdominal volume index in females could be used to identify risk for metabolic syndrome in the transition from adolescence to adulthood.

This Special Issue also includes four intervention studies, which focused on the prevention or treatment of childhood obesity [14,15,16,17]. Zhu et al. implemented a school-based intervention to decrease the consumption of sugar-sweetened beverages (SSBs) among children and adolescents from China. The intervention was designed based on the ecological model, targeted multiple levels for behavioral change (individuals, families, peers and school) and was implemented over 1 year by teachers in collaboration with public health doctors at the local community health centers. The authors recorded significant reductions in both the frequency and the quantity of SSBs consumed in the intervention compared to the control group, especially in the elementary schools and in boys. Furthermore, the study by Lubrecht et al. examined the effectiveness of a lifestyle intervention in children aged 2–18 years with severe obesity. More specifically, the authors adopted a longitudinal design to compare the effects of a standardized intervention between younger children (2–12 years old) and adolescents (13–18 years old) over a period of 2 years. The intervention was delivered by a multidisciplinary team, consisting of pediatricians, dieticians, psychologists, pedagogues, physical activity coaches and nurses, and included a personalized approach based on the children’s/adolescents’ characteristics and needs. The findings showed that the BMI z-scores tended to decrease more in children compared to in adolescents over time. The intervention led to significant improvements in participants’ cardiometabolic indices (e.g., lipid profiles, the levels of glucose metabolism indices, and alanine aminotransferase), indicating that lifestyle modifications in severe obesity can produce clinically significant improvements in children’s’/adolescents’ weight status and health. The study by Hawkins et al. implemented the “Healthy Schoolhouse 2.0 program” in elementary schools in Washington, DC, USA, over a 5-year period. This study was specifically designed to provide equitable access to the intervention for the participants and aimed to increase their nutrition literacy. During follow-up, an increase in students’ nutrition knowledge scores was observed in the intervention group, especially among students whose teachers delivered three nutrition lessons compared to those who implemented fewer. Finally, Jones et al. present the design of a novel dietary intervention (RCT) in women with gestational diabetes, which aims to prevent obesity among their offspring by the age of 3 years. This intervention will restrict energy intake through women’s diets, using a whole-diet replacement, and the results are expected from 2026 onwards.

The review of Motevalli et al. introduced an “Etiology-Based Personalized Intervention Strategy Targeting Childhood Obesity” (EPISTCO) model, which provides a guide to healthcare professionals (HCPs) working with children to better understand the determinants of childhood obesity and then deliver tailormade interventions according to children’s and adolescents’ personalized characteristics and needs [18]. In this context, several biological, behavioral and environmental factors are assessed, and the individual’s barriers and facilitators for the adoption of healthy lifestyle behaviors are identified. The novelty of this model is that it includes a four-step, multicomponent intervention, which foresees the personalization of the whole process by the multidisciplinary team of HCPs, thus potentially increasing the effectiveness of the intervention.

The systematic review by Leme et al. focused on obesity-prevention strategies in adolescence and compared the effectiveness of two types of interventions: those targeting energy balance (decreasing energy intake and/or increasing energy expenditure) and those aiming to reduce disordered eating behaviors to promote a positive food and eating relationship [19]. Both types of studies demonstrated poor clinical outcomes, with the interventions focusing on energy balance failing to support adolescents in maintaining the positive changes in their lifestyle behaviors and weight status achieved during their implementation, while the second group of interventions were not effective in reducing weight over time. Considering that the interventions aiming to reduce disordered eating behaviors were effective in reducing adolescents’ body dissatisfaction, dieting and weight-control behaviors, the authors suggested that new studies in this field needed to be considered and designed accordingly to achieve weight loss.

Another study by Flores-Ramírez et al. reviewed the combined effects of l-citrulline supplementation and aerobic training on vascular function in different age groups [20]. The findings of this study provide evidence to support the implementation of moderate-to-high-intensity interventions (duration: 12–32 weeks) for improving indices of vascular function and cardiovascular risk factors. Moreover, l-citrulline supplementation seems to be effective in improving children’s and adults’ nitric-oxide levels and the bioavailability of l-citrulline, which plays a pivotal role in nitrogen homeostasis and improves various cardiovascular risk factors in adults (especially in those with obesity). Given these results, the authors suggested that new interventions aiming to examine the combined effects of l-citrulline supplementation and aerobic training on the vascular function of children and/or young adults living with obesity and/or impaired metabolic profiles are needed to address the negative impact of obesity on health in the early stages of life.

In conclusion, the results of the studies included in the Special Issue “Childhood Obesity: Nutrition and Lifestyle Determinants, Prevention and Management” of the journal Nutrients show high rates of childhood obesity; provide new insights regarding the use of novel methods in identifying impaired cardiometabolic profiles in children with obesity, as well as on the role and interplay of its determinants; and present new interventions and models for the prevention or treatment of obesity in children and adolescents. With the hope that this new evidence will open new horizons in the field of childhood obesity and will further improve the healthcare process in pediatric health, the Guest Editors would like to thank all the contributors for the publication of this Special Issue.
